# A Multiplexed Device Based on Tunable *Nanoshearing* for Specific Detection of Multiple Protein Biomarkers in Serum

**DOI:** 10.1038/srep09756

**Published:** 2015-05-15

**Authors:** Ramanathan Vaidyanathan, Lara Michelle van Leeuwen, Sakandar Rauf, Muhammad J. A. Shiddiky, Matt Trau

**Affiliations:** 1 Centre for Personalised Nanomedicine, Australian Institute for Bioengineering and Nanotechnology (AIBN), Corner College and Cooper Roads (Bldg 75), The University of Queensland, Brisbane QLD 4072, Australia; 2 School of Chemistry and Molecular Biosciences, The University of Queensland, Brisbane, QLD 4072, Australia

## Abstract

Microfluidic flow based multiplexed devices have gained significant promise in detecting biomarkers in complex biological samples. However, to fully exploit their use in bioanalysis, issues such as (i) low sensitivity and (ii) high levels of nonspecific adsorption of non-target species have to be overcome. Herein, we describe a new multiplexed device for the sensitive detection of multiple protein biomarkers in serum by using an alternating current (ac) electrohydrodynamics (ac-EHD) induced surface shear forces based phenomenon referred to as *nanoshearing*. The *tunable* nature (via manipulation of ac field) of these *nanoshearing* forces can alter the capture performance of the device (*e.g.*, improved fluid transport enhances number of sensor-target collisions). This can also selectively displace weakly (nonspecifically) bound molecules from the electrode surface (*i.e.*, fluid shear forces can be tuned to shear away nonspecific species present in biological samples). Using this approach, we achieved sensitive (100 fg mL^−1^) naked eye detection of multiple protein targets spiked in human serum and a 1000-fold enhancement in comparison to hydrodynamic flow based devices for biomarker detection. We believe that this approach could potentially represent a clinical diagnostic tool that can be integrated into resource-limited settings for sensitive detection of target biomarkers using naked eye.

The complexity and heterogeneity associated with diseases such as cancer require the measurement of multiple biomarkers to provide a more comprehensive understanding of the biological process and disease dynamics^1^. In resource-constrained settings, a simple methodology for the routine assessment of multiple molecular biomarkers promises to transform disease diagnosis by providing clinically useful information^2^,^3^. In healthcare settings, the integration and utilization of cancer biomarkers is therefore largely dependent on the development of a simple, rapid and multiplexed platform with the ability to detect multiple targets with high specificity and sensitivity^4^. In recent years, a wide range of assay formats have been developed for the measurement of multiple proteins using enzyme-linked immunosorbent assay (ELISA)^5^,^6^, nanomaterials (*e.g.*, nanotubes^7^, nanowires^4^), electrochemical immunosensors^8^ and mass spectrometry^9^. In addition, a number of microfluidic approaches based on chemically modified sensors surfaces relying on diffusive mixing (*i.e.*, increased number of sensor-target collisions) of targets have also been developed to enhance the capture performance of the devices^10^,^11^. Despite their impressive performances, their integration into resource-limited settings that require simple on-site diagnosis to improve treatment and survival rates, is restricted due to the need for sophisticated detection procedures and operational control systems.

Over the past decade, several labeling and signal enhancement strategies have been developed that enabled rapid on-site detection of protein biomarkers using chemiluminescence, colorimetric and optical read-out methods^6^,^12^. Most of these methods are based on conventional immunoassays that rely on an antibody-functionalized solid support for target capture and a reporter antibody for assay read-out. One of the major limitations of these technologies is the nonspecific adsorption of non-target species (molecules, cells, etc) onto to the sensor surface^13^. Generally, concentrations of non-target species present in biological fluids (serum, bloods, plasma, etc) are several orders of magnitude higher than that of target proteins (10^−16^–10^−12^ M). These non-target species tend to simply adhere to the sensor surface without any specific receptor (*e.g.*, antibody) recognition, thereby limiting the sensitivity and specificity of detection^14^. In recent years, much attention has been given towards the use of antifouling layers or coatings to minimize the nonspecific adsorption of molecules onto the sensor surface^15^.

Herein, we developed a microfluidic based multiplexed device that can address these issues associated with traditional immunoassay systems such as (i) low sensitivity and (ii) high levels of nonspecific adsorption. The device contains three parallel channels, each of which contains a long array of planar asymmetric electrode pairs (see Experimental for details of the electrode design). The working principle of the device relies on the use of ac-EHD induced *nanoshearing* forces that are generated within nanometer distances of the electrode surface and can engender lateral fluid flow towards the direction of the broken asymmetry^16^,^17^,^18^. The resulting fluid mixing (*i.e.*, due to the ac-EHD induced flow) around the sensor surfaces can significantly enhance capture efficiency due to increased number of sensor-target collisions (Fig. 1). Additionally, the *tunable* nature of these forces (*e.g.*, *via* the application of an ac electric field) enable the preferential selection of strongly (specifically) bound proteins over more weakly (nonspecifically) bound proteins on the sensor surfaces. We recently demonstrated the use of surface shear forces in a device containing planar and three-dimensional (3D) microtip electrodes for capture and detection of protein biomarkers using fluorescence based detection methods^17^. In this study, we have used for the first time, a multiplexed device for simultaneous naked eye on-chip detection of multiple target proteins. To demonstrate the capture performance and multiplexed analysis of protein biomarkers, the electrode surface on individual channels of the device was initially functionalized (Fig. 2) with capture antibodies specific to human epidermal growth factor receptor 2 (HER2), prostate specific antigen (PSA) and immunoglobulin (IgG) proteins. Serum samples containing target proteins (*e.g.*, HER2, PSA and IgG) were run through the devices under the determined optimal ac-EHD field. The captured proteins were detected via a rapid (~5 min) on-chip naked eye read-out obtained using the catalytic oxidation of 3,3',5,5'-Tetramethylbenzidine (TMB)^19^. The corresponding absorbance measurements obtained using UV-Visible spectroscopy measurements (maximum absorbance at 652 nm; *A*_652 nm_) provided quantitative information on the captured proteins.

## Results and Discussion

### Device design and mechanism

The controlled mixing of fluid at a microscopic level (*e.g.*, fluid micromixing) is of particular importance in a heterogeneous microfluidic system designed to capture and detect low concentration of molecular analytes^20^. Microfabrication techniques enable the creation of near limitless geometric designs of microfluidic devices which engender localized fluid micromixing and consequently enhance analyte transport under pressure driven fluid flow (*i.e.*, hydrodynamic flow)^21^,^22^. Most of these devices are limited by their ability to only manipulate fluid micromixing by adjustment of macroscopic flow rate (via a syringe pump) or through predesigned micro-geometries of microfluidic devices. As an alternative to these devices, we propose the use of *tunable* ac-EHD induced fluid shear forces (*e.g.*, fluid *nanoshearing*) to achieve better control on fluid flow. In order to investigate the utility of ac-EHD forces as an effective way to manipulate fluid flow and concomitant fluid micromixing (*i.e.*, enhanced transport of analytes) within the flow channel, we fabricated a multiplexed microfluidic device (see Methods for design and fabrication details; Fig. 3a,b and Supplementary Fig. S1,S2) comprising of three independent microfluidic channels each containing an array of 100 asymmetric electrode pairs. The critical gap (*r_0_*) between small (*d_1_*) and large electrodes (*d_2_*) in each pair and the distance between adjacent electrode pairs (*r_1_*) in this design will determine the ac-EHD induced fluid flow and concomitant micromixing^23^,^24^. It has previously been predicted that electric field induced fluid flow rate is inversely proportional to the geometry of the electrodes within a microchannel^23^. In our case, the ratios of these critical determinants were maintained uniformly throughout each channel as: *r*_0_/*d*_2_ = 0.2, *r*_1_/d_2_ = 0.6, *d*_1_/*d*_2_ = 0.2, respectively. While traditional microfluidic devices rely on a pressure-driven system (*e.g.*, via an external syringe pump) for fluid flow, we believe that the asymmetry in electrode geometry in our device is sufficient enough to produce highly effective ac-EHD induced fluid flow within the channel (*i.e.*, acts as a fluid pump).

**Figure 1:**
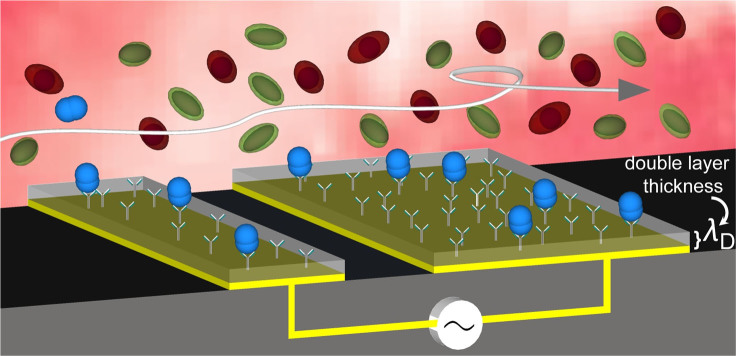
Schematic representation of ac-EHD induced *nanoshearing*. The application of an ac field (*e.g.*, alternating potential difference) across each electrode pair induces charges on the electrode surface. The lateral variation in number of induced charges and their spatial distribution gives rise to non-uniform local forces on the large and small electrode with the resultant force giving rise to lateral unidirectional flow towards the larger electrode. Since all the shear forces are contained within nanometers (*λ*_D_ = double layer thickness) of an electrode surface, these forces engender fluid flow to increase the number of target-antibody (blue) collisions whilst shearing away nonspecifically bound molecules (green and red).

Fig. 1 illustrates the mechanism of ac-EHD induced fluid flow. The small and large electrodes within each microchannel act as the cathode and anode respectively (see Fig. 3c for experimental setup). Upon the application of an ac electric field, (*i.e.*, potential difference), the asymmetric geometry of the electrodes in the pair give rise to a lateral variation in the amount of induced (double layer) charges and spatial distribution of the charges on the electrode surface^23^. The induced charges in the double layer of each electrode experience a force due to interaction of the tangential component of non-uniform field. Under electric field, the induced charges on the larger electrode generate stronger lateral forces than those on the smaller electrodes, resulting in a fluid flow towards the larger electrode in the pair (Fig. 1). Notably, the generated lateral forces are the product of charge and electric field (i.e., F = ρE) and thus, reversing the polarity of the field also results in a steady flow towards the larger electrode. A special feature of this flow is that because all of the *induced charges* occur only within the double layer of the electrode (the characteristic thickness of the double layer can be estimated by considering Debye Hückle approximation and use of equation given in Refs. 17, 25), all of the ac-EHD forces on the fluid also occur strictly within this region. Critically, due to the solution ionic strengths used in this study (1 mM phosphate buffer saline (PBS)) the electrical double layers are typically on the order of 3 to 4 nm in size, meaning this system engenders fluid shear forces and concomitant fluid mixing within few (<5 nm) nanometers of an electrode/solution interface. We believe this is one of the most fascinating features of this phenomenon and have therefore termed the ability to tune these fluid shear forces at the nano-scale as *nanoshearing*, given the capacity of these shear flows to displace weakly bound molecules on the surface.

**Figure 2:**
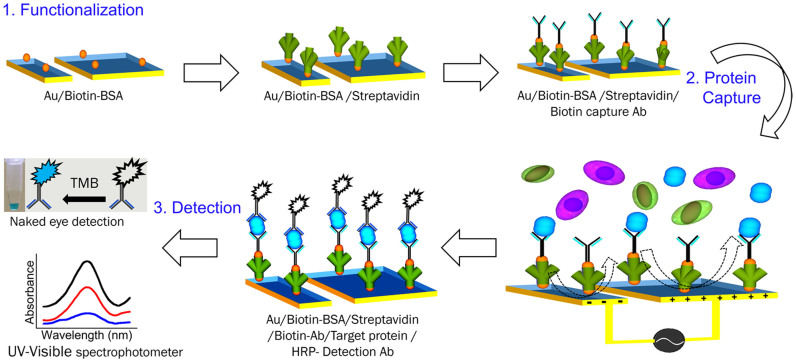
Capture and detection of protein biomarkers. Schematic representation of device functionalization, protein biomarker capture, and colorimetric detection of captured proteins.

To investigate the applicability of this phenomenon for protein biomarker detection, all channels in the device were functionalized with anti-HER2 (see Methods and Fig. 2 for protein capture and detection) capture antibody. Serum samples spiked with (100 pg mL^−1^) and without HER2 antigen were then driven through the channels under an applied ac-EHD field strength of *f* = 1 kHz and *V*_pp_ = 100 mV. This step was then followed by the addition HRP conjugated secondary antibody solution. Upon addition of TMB, a visual color change to deep blue was observed due to the catalytic oxidation of TMB, allowing rapid colorimetric readout for naked eye detection of captured proteins. Subsequently, absorbance measurements (maximum absorbance at 652 nm; *A*_652 nm_) of these colorimetric solutions were used to obtain quantitative information on the captured protein biomarkers (see Methods for details).

Colorimetric readouts provide two key advantages over traditional microscopic readouts that include: (i) minimal instrumental requirements (*e.g.*, does not require expensive instruments such as microscope, light scattering equipments) for analysis and (ii) rapid naked-eye readout which is suitable for on-site monitoring of disease biomarkers and subsequent quantitative information on the molecule of interest (*e.g.*, light transmitted is proportional to concentration of target) improve the accuracy of analysis. Under applied ac-EHD field, the color change to deep blue (Supplementary Fig. S3a, inset) and its corresponding absorption spectra (black; Supplementary Fig. S3a), suggested high capture levels of HER2 proteins in serum. In case of serum samples without HER2, negligible nonspecific binding of the FITC conjugated detection antibody was observed under ac-EHD field (blue; Supplementary Fig. S3a). Representative fluorescence images (see Methods for details) of the detected HER2 protein and nonspecifically bound detection antibody are shown in Supplementary Fig. S3b, c. These data suggests that ac-EHD induced fluid *nanoshearing* is applicable for protein biomarker detection due to the effective stimulation of fluid flow and concomitant fluid mixing around the functionalized electrodes thereby resulting in high capture levels.

### Effect of applied ac-EHD force on protein capture

ac-EHD induced fluid flow within a given device is dependent on a number of key experimental parameters that include (i) applied frequency, (ii) amplitude, and (iii) electrolyte conductivity^26^,^27^. Because the capture performance of our devices is dependent on ac-EHD induced surface shear forces (*i.e.*, fluid flow), it is important to determine the optimal fluid flow conditions (*e.g.*, ac-EHD field). To optimize the ac-EHD induced forces for protein biomarker capture and detection, serum samples containing spiked target HER2 antigen (100 pg mL^−1^) were driven through the antibody-functionalized devices (*e.g.*, all three channels were functionalized with anti-HER2 antibody) under frequency (*f*) range of 600 Hz-100 kHz at constant amplitude (*V*_pp_) of 100 mV (Fig. 4a, b). Initially, a sharp color change to deep blue was observed within the frequency range of 600 Hz to 1 kHz. At the frequency range of 1 kHz to 100 kHz, a gradual color change from deep blue to a more clear solution was observed. These data indicate that the capture performance of the device is a function of the applied frequency. The corresponding UV-Vis measurements (Fig. 4b, inset) and absorbance peak at 652 nm (*A*_652 nm_; Fig. 4b) corroborate with these observations as evident from the similar trend in capture levels. This variation in capture levels with the applied frequency is presumably due to the manipulation of shear forces within the double layer of the functionalized electrodes (*e.g.*, capture domain). At frequencies < 1 kHz, the stimulation of fluid flow was not high enough to allow effective collisions between the antibody-modified surface and specific proteins, and thus resulting in low capture efficiency. At the frequency of 1 kHz, the stimulation of fluid flow around the sensor surface was optimal to create effective sensor-target collisions, resulting in maximum capture efficiency. This frequency also offers high enough surface shear forces that can effectively remove non-specifically adsorbed species from the surface. However, with the increase in applied frequency this stimulation of fluid flow around the sensor surface was found to be strong enough to wash away nonspecific species along with the specific targets (a condition where shear forces > antibody-target affinity interaction), thereby decreasing the capture performance of the device.

### Analytical performance of ac-EHD device

To assess accuracy of immunocapture, control experiments were performed using (i) devices functionalized (*e.g.*, all three channels) without anti-HER2 capture antibody and (ii) devices without FITC conjugated anti-HER2 detection antibody (*e.g.*, all three channels). Serum samples spiked with HER2 (100 pg mL^−1^) were driven through the devices using the field strength of *f* = 1 kHz at *V*_pp_ = 100 mV. In both cases, negligible background response due to the nonspecific binding of low levels of detection antibody (*e.g.*, device without capture antibody; Supplementary Fig. S4a) or peroxidase (*e.g.*, HRP) (*e.g.*, device without detection antibody; Supplementary Fig. S4b), were observed. This high level of specificity and accuracy of immunocapture in our method is presumably due to the use of ac-EHD induced *nanoshearing* that can remove nonspecifically bound biological species (in this case, detection antibody and peroxidase (*e.g.*, HRP) for TMB oxidation). Further, to investigate the presence of any background signal from the BSA blocked PDMS, control experiments were performed with incubation of HRP conjugated detection antibody post streptavidin attachment. As can been seen in Suppplementary Fig. S4c, negligible background response was observed indicating the low level of nonspecific binding of biomolecules onto BSA blocked PDMS surfaces. This suggests that the detected response was primarily from biomolecules on the electrode surface rather than any passivation onto the PDMS channels.

To investigate the dynamic range and lower limit of detection (LOD) of this device designated concentrations of HER2 (1 ng mL^−1^ to 10 fg mL^−1^) were spiked in serum and the samples were run on anti-HER2 functionalized (*e.g.*, all three channels) devices under ac-EHD (*f* = 1 kHz and *V*_pp_ = 100 mV) flow conditions (Fig. 5a). Individual devices were used for each designated concentration and three individual trials (*e.g.*, three channels) were performed on each device. Corresponding absorbance spectra is given in Fig. 5b. Under the applied ac-EHD force, the device was sensitive to detect 100 fg mL^−1^ HER2 antigen spiked in serum samples. The correlation in signal enhancement with increase in protein concentration was found to be R^2^ = 0.939. Furthermore, the reproducibility of detection was determined from three separate trails (*e.g.*, three separate functionalized channels) and resulted in a %RSD of <4.7% (*n* = *3*). This level of sensitivity and reproducibility is comparable to protein detection technologies based on 3D microelectrodes^17^, redox cycling^28^, enzyme based signal amplification methods^29^ and ac electrokinetic enrichment^30^,^31^ of protein biomarkers. However, these methods requiring sophisticated detection procedures and operational conditions are not ideally suited for analysis in remote settings that require minimal operational requirements. In contrast, the use of an ac-EHD induced surface based phenomenon to mediate the target capture process and naked eye read-out (followed by UV-Vis measurements) can quantify the presence of target analytes at concentrations that are probably undetectable using standard ELISA or polymerase chain reaction^32^.

Pressure driven flow (*e.g.*, hydrodynamic flow) based immunoassay devices have previously been developed for the detection of protein biomarkers^33^,^34^. In these systems, external syringe pumps are used to deliver necessary fluids in these systems. However, the use of pressure-driven flow based systems has two key limitations in comparison to ac-EHD induced fluid flow that include (i) hydrodynamic flow follows a parabolic flow profile that has a stationary boundary layer of fluid at the solid-liquid interface^35^, resulting in the inability to precisely manipulate shear forces on the electrode/solution interface, and (ii) external syringe pumps cannot be incorporated into portable systems that require on-chip fluid pumping involving no moving parts^26^,^27^. Thus, to investigate this, we then compared the capture device performance under ac-EHD flow to that under a hydrodynamic flow. Hydrodynamic flow based devices (see Methods) operated under a pressure-driven system (*via* a syringe pump) at a flow rate (8 μL min^−1^) similar to that observed under ac-EHD flow conditions (*f* = 1 kHz and *V*_pp_ = 100 mV). Samples containing designated concentrations of HER2 (1 ng mL^−1^ to 10 fg mL^−1^) spiked in serum were run on anti-HER2 functionalized devices under ac-EHD and pressure driven flow conditions. Under the applied ac-EHD force, colorimetric readouts (Supplementary Fig. S5a–c, inset) and corresponding absorbance measurements (Supplementary Fig. S5a–c) suggested an approximately 1000 fold (Supplementary Fig. S5d; 100 fg mL^−1^ (ac-EHD; black) *versus* 100 pg mL^−1^ (pressure driven flow; red)) increase in detection capabilities in comparison to the pressure drive flow based devices. Furthermore, absorbance peak at 652 nm (*A*_652 nm_; Supplementary Fig. S5d) suggested an approximately 5-fold increase in capture level at all concentrations under ac-EHD in comparison with pressure driven flow based controls. This enhancement in HER2 capture with the use of ac-EHD forces in comparison to pressure driven flow based devices could possibly be due to the effective stimulation of fluid mixing (*e.g.*, *nanoshearing*) for enhanced sensor-target collisions as well as the ability to remove nonspecifically adsorbed molecules from the electrode surface.

### Multiplexed protein detection using ac-EHD device

To demonstrate the utility of our device for multiplexed protein biomarker detection, individual channels of the device was functionalized (Fig. 6a) with anti-HER2 (*e.g.*, channel-1), anti-PSA (*e.g.*, channel-2) and anti-IgG (*e.g.*, channel-3) capture antibody. Serum samples spiked with target proteins (100 fg mL^−1^) along with large excess of two non-target proteins (1 ng mL^−1^ for both cases) were driven through the channels under the frequency of *f* = 1 kHz at *V*_pp_ = 100 mV. For instance in channel-1, samples containing HER2 (100 fg mL^−1^) target antigen spiked along with non-target PSA (1 ng mL^−1^) and IgG (1 ng mL^−1^) proteins were driven through the channel under ac-EHD flow conditions. Similarly for channel-2 and 3, PSA (channel-2) and IgG (channel-3) target antigens were spiked in serum along with HER2 (channel-2,3) and IgG (channel-2) or PSA (channel-3), respectively. Fig. 6b demonstrates multiplexed protein biomarker detection under the ac-EHD flow conditions of *f* = 1 kHz at *V*_pp_ = 100 mV. Clearly, it was evident that the device was sensitive enough to detect low concentration of target proteins in the presence of a large excess of non-target proteins in serum (10^4^ fold higher concentrations than target proteins). The performance of the device was also reproducible for the capture and detection of HER2, PSA and IgG biomarkers with an inter-assay (*e.g.*, from individual functionalized channels) RSD of 10.3% (*n* = *3*).

Furthermore, we also validated the specificity of capture at such low concentrations by performing additional control experiments on anti-HER2 functionalized devices to determine level of background response from (i) serum samples spiked only with non-target proteins (PSA and IgG; 1 ng mL^−1^) and (ii) only serum (*i.e.*, without target and non-target proteins). Under the applied field strength of *f* = 1 kHz at *V*_pp_ = 100 mV, a very low background response from the non-target proteins (black; Supplementary Fig. S6) and/or detection antibody (blue; Supplementary Fig. S6) was observed. This level of background response indicates that the immunocapture was highly specific and also suggests that our device is applicable for the detection of multiple protein biomarkers from complex biological samples. The device performance is comparable to a number of multiplexed detection platforms using bio-barcode assays^36^, nanoparticle based immunocapture^37^, nanowire sensors^4^, nanostrucutred microfluidic chips^38^ and nucleic acid identifiers^39^, respectively. Although this level of detection from complex biological samples is believed to be significant and relevant to clinical applications^40^, we believe that further optimization to the geometric arrangement of electrodes, device design and ac-EHD parameters could possibly further enhance the detection capabilities of the device.

In conclusion, we have developed a simple method for the simultaneous detection of multiple protein biomarkers by utilizing the capability to manipulate surface shear forces (*e.g.*, *nanoshearing*) via the applied ac-EHD field. The unique features of this method lies in the (i) effective use of *nanoshearing* to enhance target capture levels and displace nonspecific proteins from electrode surfaces, (ii) use of simple on-chip naked eye readouts that is potentially suited for biomarker analysis in resource-limited settings, and (iii) the use of a long array of gold microelectrode pairs both as the capture domain and fluid pump (avoids use of additional pumps, valves, etc). We have demonstrated the ability of this approach to enhance the detection capabilities of the device by upto 1000 fold in comparison to hydrodynamic flow based devices and also sensitively detect multiple protein biomarkers at concentrations as low as 100 fg mL^−1^ in human serum. We believe that this approach can potentially be applicable for any diagnostic biochemical assay in resource-constrained settings. Furthermore, we propose that this device can also be integrated into a various clinical and biological settings.

## Methods

### Reagents

All general-use reagents were purchased from Sigma Aldrich (Australia) and immunoassay reagents were obtained from R&D/Life Technologies (Burlington, ON), Thermo-Fisher Scientific (Australia), Abcam (Australia) and Invitrogen (Australia). All reagents and washing solutions used in the experiments were prepared using phosphate buffer saline (PBS, 10 mM, pH 7.4). Photoresists for fabrication (MicroChem, CA) were used as per manufacturer's instructions.

### Design of multiplexed ac-EHD device

**Figure 3:**
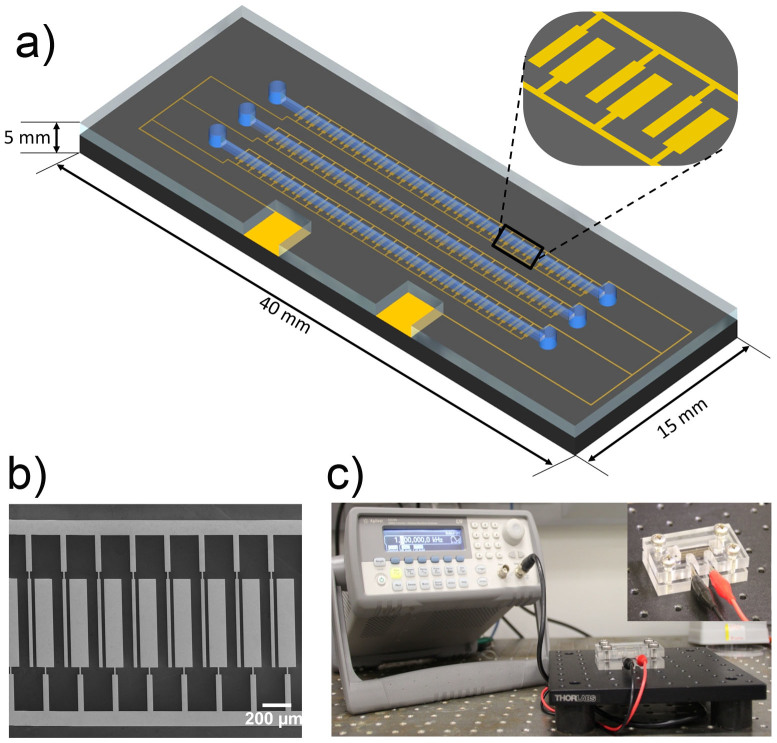
Multiplexed *nanoshearing* device. (a) Schematic of a multiplexed microfluidic device for protein biomarker detection comprising of three independent channels. (b) Corresponding scanning electron microscopy (SEM) image of enlarged segment of the device. (c) Experimental setup for protein biomarker detection using a multiplexed ac-EHD device. Inset shows the device sandwiched between custom build holders with ac signal being supplied using a signal generator.

**Figure 4:**
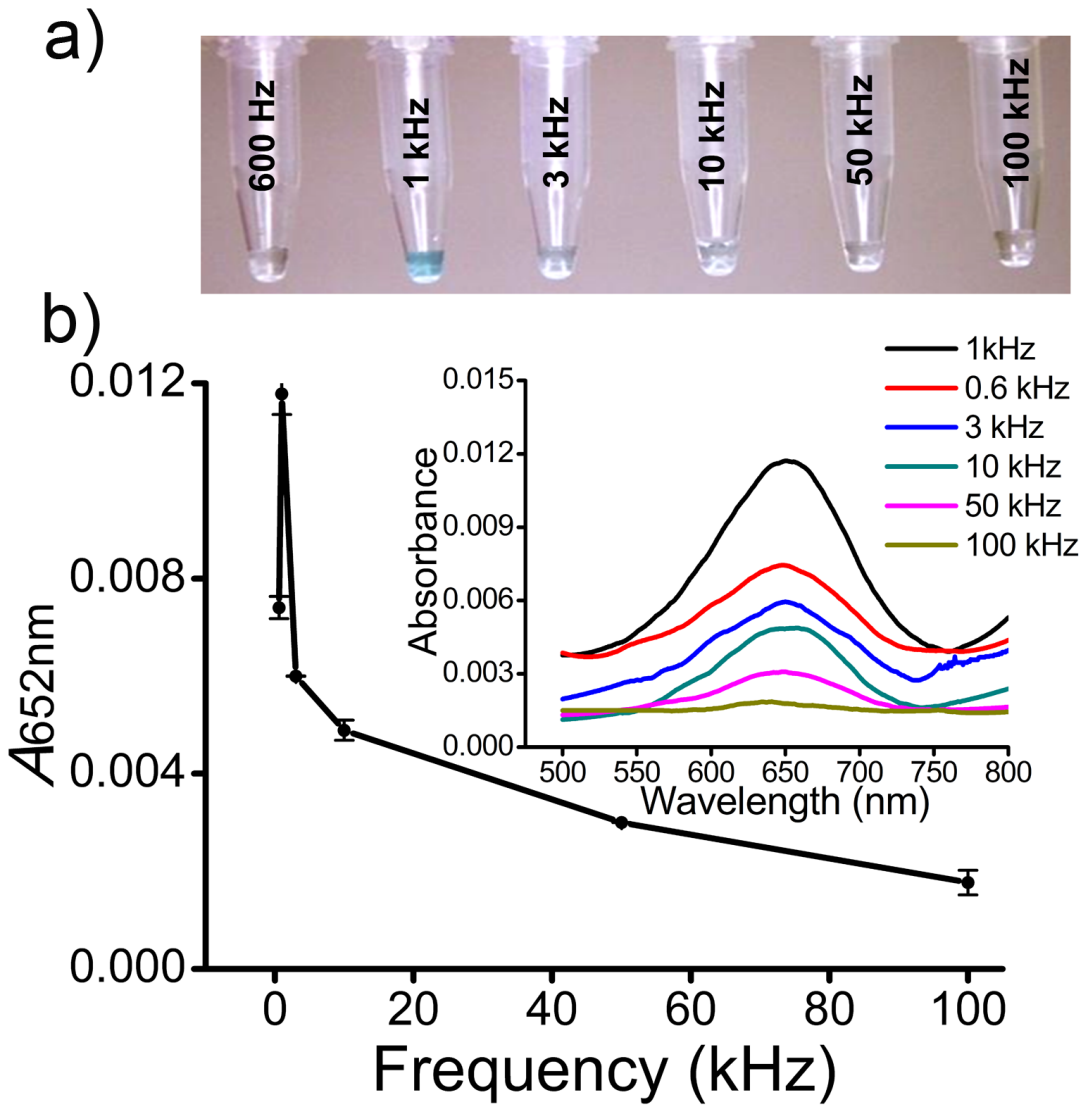
Effect of frequency on protein capture. (a) Colorimetric detection of HER2 antigen (100 pg mL^−1^) spiked in serum under the frequency range *f* = 600 Hz- 100 kHz at *V*_pp_ = 100 mV. (b) Absorbance peak at 652 nm (*A*_652 nm_) for HER2 (100 pg mL^−1^) spiked in serum under the frequency range *f* = 600 Hz- 100 kHz at *V*_pp_ = 100 mV. Each data point represents the average of three separate trials (*n* = *3*) and error bars represent standard error of measurements within each experiment. Inset shows corresponding UV-Vis absorption spectra obtained from respective colorimetric solutions.

**Figure 5:**
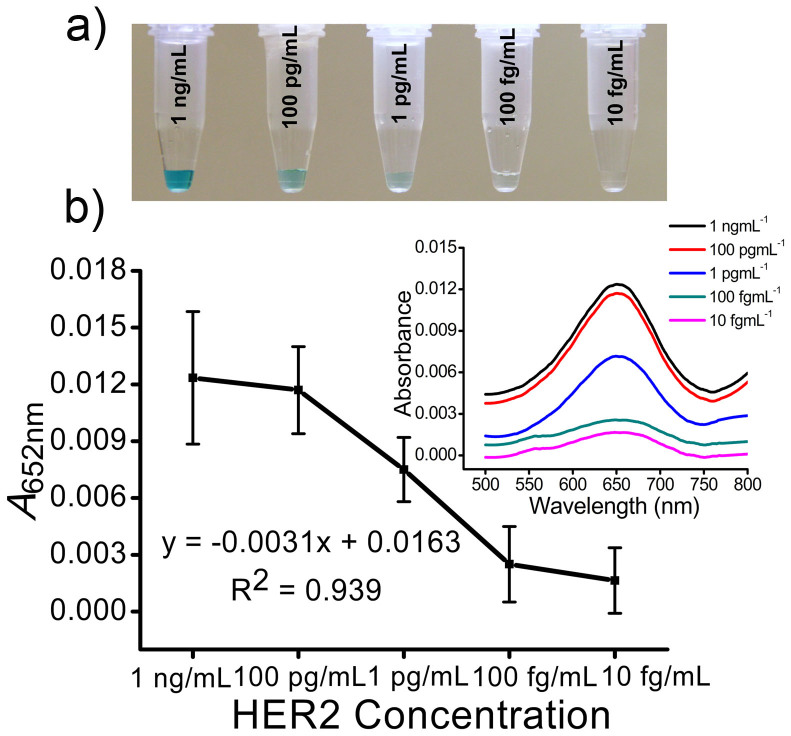
Sensitivity of detection. (a) Colorimetric detection of HER2 protein (1 ng mL^−1^ to 10 fg mL^−1^) spiked in human serum under ac-EHD field strength of *f* = 1 kHz at *V*_pp_ = 100 mV. (b) Absorbance peak at 652 nm (*A*_652 nm_) for serum samples containing spiked HER2 protein (1 ng mL^−1^ to 10 fg mL^−1^) under ac-EHD field strength of *f* = 1 kHz at *V*_pp_ = 100 mV. Each data point represents the average of three separate trials (*n* = *3*) and error bars represent standard error of measurements within each experiment. Inset shows corresponding UV-Vis absorption spectra obtained from respective colorimetric solutions.

**Figure 6:**
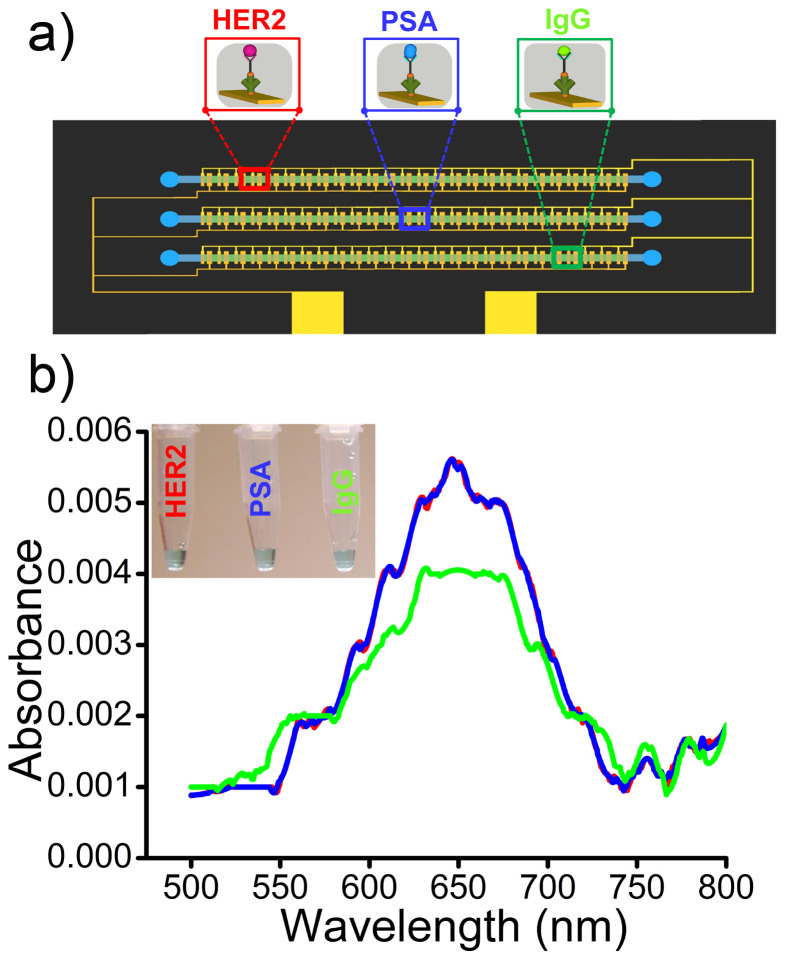
Detection of multiple protein biomarkers. UV-Vis absorption spectra of serum samples spiked with non-target proteins (1 ng mL^−1^) along with target proteins (100 fg mL^−1^) under the ac-EHD field strength of *f* = 1 kHz at *V*_pp_ = 100 mV. Individual channels of the device were functionalized with specific capture antibody to detect target HER2 (red; channel-1), PSA (blue; channel-2) and/or IgG (green; channel-3) proteins, respectively. Inset shows naked eye detection of the detected target protein in each channel of the device under the applied ac-EHD field strength.

We designed a multiplexed microfluidic device containing three indepedent microchannels with individual inlet and outlet ports (Supplementary Fig. S1 and Fig. 3a). Each channel comprehends 100 planar asymmetric microelectrode pairs with the small and large electrodes measuring 25 μm and 125 μm in width, respectively. The small and large electrodes are separated by a distance of 25 μm and adjacent electrode pairs in each segment are separated by a distance of 75 μm. The small and large electrodes of each asymmetric electrode pair present in all three channels are connected to two individual gold connecting pads that act as cathode and anode (or vice versa) of an electrolytic cell (Supplementary Fig. S1). The device design was made using Layout Editor (L-edit V15, Tanner research Inc., CA) and written to a chrome mask (5 × 5 in.; Qingyi Precision Mask-Making (Shenzhen) Ltd., China).

### Fabrication of devices

Devices were fabricated at the Queensland node of Australian National Fabrication Facility (Q-ANFF node). Fabrication of the device involves a two-step photolithographic process as outlined in Supplementary Fig. S2. To fabricate asymmetric gold electrode patterns, an initial passivation layer of silicon oxide (thickness, 300 nm) was deposited on silicon wafers (diameter, 100 mm; thickness, 1.0 mm; single-side polished) in an oxidation furnace. The wafers were then cleaned with sonication in acetone for 5 min, rinsed with isopropyl alcohol and water for another 2 min, and dried with the flow of nitrogen. A thin film of negative photoresist (AZnLOF 2070, MicroChem, Newton, MA) was spin coated (3000 rpm for 30 s) onto the wafer, followed by a soft baking step at 110°C for 6 min. Subsequent UV exposure (280 mJ/cm^2^) using a mask aligner (EVG 620, EV Group GmbH, Austria) was followed by a short post-bake step at 110°C for 3 min. Development using AZ 726 developer for 3 min revealed the asymmetric electrode patterns. Metallic layers of Ti (10 nm) and Au (200 nm) were deposited using an electron beam (e-beam) evaporator (Temescal FC-2000) under high vacuum conditions followed by lift-off using ethanol revealed the patterned gold electrodes. The wafers were then diced (ADT 7100 wafer precision dicer) to obtain individual devices. Devices were characterized by SEM analysis using a JEOL (model 6610) instrument operating at an accelerating voltage of 10 kV.

To fabricate the microfluidic channels, a layer of negative photoresist (SU-8 2150; MicroChem, Newton, MA) was spin coated at 1800 rpm onto a clean silicon wafer to generate masters (Supplementary Fig. S2) for the three independent microfluidic channels (width, *w* = 400 μm; height, *h* = 300 μm; length, *l* = 25 mm) with 1.0 mm diameter inlets and outlet ports. Soft and hard baking steps were performed as per manufacturer's instructions. Briefly the wafer was soft baked through a series of step change in temperature (65°C for 7 min → 95°C for 60 min → 65°C for 5 min). Subsequent UV exposure (380 mJ/cm^2^) was followed by a post-bake step (from 65°C for 5 min → 95°C for 20 min → 65°C for 3 min) and development in propylene glycol methyl ether acetate (PGMEA) for 45 min revealed the fluidic channels. The masters were then used as molds, on which polydimethylsiloxane (PDMS) prepolymer mixed with its crosslinker (ratio 10:1; Sylgard 184 kit, Dow Corning) was poured, degassed, and allowed to cure in a conventional oven at 65°C for 2 h. The cured PDMS replicas were removed from the molds and 1 mm holes were punched into PDMS at either ends of the channel to define the inlets and outlet ports (diameter, 1.0 mm).

### Device functionalization

Gold electrodes were cleaned with sonication in acetone for 5 min, rinsed with isopropyl alcohol and water for another 2 min and dried with the flow of nitrogen. Following this, the array of gold microelectrode pairs within the capture domain of the channel was then modified with the capture antibody using avidin-biotin chemistry (Fig. 2) in a three step process that involves (*i*) an initial incubation in biotinylated BSA (200 μg mL^−1^ in PBS, Invitrogen) solution for 2 h, (*ii*) followed by coupling with streptavidin (100 μg mL^−1^ in PBS, Invitrogen) for 1 h at 37°C and (iii)streptavidin conjugated channels were then coated with biotinylated antibodies (10 μg mL^−1^ of biotinylated anti-HER2, anti-PSA or anti- IgG in PBS) for another 2 h. Channels were flushed three times with PBS (10 mM, pH 7.0) to remove any unbound molecules after each step. Each of the surface modification steps (*e.g.*, biotinylated BSA, streptavidin, and capture antibody) was performed manually by filling the microchannel with corresponding solution to specifically modify the array of gold electrodes within the capture domain. PDMS was bonded to the devices and sandwiched between custom build holders (Fig. 3c) for samples to be filled and withdrawn via inlet and outlet ports respectively. Prior to bonding PDMS with the devices, they were blocked with 3% BSA overnight to avoid nonspecific adsorption of biomolecules during functionalization and biomarker capture. This prevented any passivation of biomolecules onto the PDMS channels.

### Protein capture and detection

The cathode and anode (small and large electrodes within the long channel) of the ac-EHD devices (Fig. 3c and Fig. S1) were connected to a signal generator (Agilent 33220A Function Generator, Agilent Technologies, Inc., CA). Serum samples were collected from healthy volunteers and stored as aliquots (80 μg μL^−1^) at −80°C upon the estimation of total protein using standard Bradford method^41^. Samples containing designated concentration of target proteins (HER2, PSA or IgG) spiked in serum was placed in the inlet reservoirs of the devices and driven through the channel by applying ac-EHD field. The ac-EHD force was applied for 30 min with 15 min intervals (without fluid flow) for a total pumping time of 2 h.

Control experiments were performed in the absence of ac-EHD field under pressure driven flow conditions using a syringe pump (PHD 2000, Harvard apparatus). FITC conjugated detection antibody (2 μg mL^−1^ of anti-HER2, anti-PSA HRP or anti-IgG in PBS) was driven through the channels under ac-EHD and/or pressure driven flow conditions. These devices were then imaged under a fluorescence microscope (Nikon eclipse Ni-U upright microscope) to obtain fluorescence images of the captured proteins. Image analysis was performed using the image processing software (Nikon Ni-S elements, Basic Research). For naked-eye detection, anti-HER2 or anti-IgG functionalized devices were then further incubated with anti-fluorescein HRP (1:1000; abcam) antibody for 30 min. The devices were flushed three times with PBS (1 mM, pH 7.0) to remove any unbound molecules. 40 μL of TMB solution was driven though the channels manually and the colorimetric reaction was allowed to proceed for 5 min to facilitate naked eye detection. The colorimetric solution was withdrawn manually from the chip using a micropipette and collected in an eppendorf for subsequent absorbance measurements. Absorbance measurements were obtained using a UV-Visible spectrophotometer (Shimadzu UV-2450, Shimadzu Corp.) and absorbance data was acquired using UVProbe (Ver. 2.3.1) data acquisition software.

## Author Contributions

M.J.A.S. and M.T. conceived the idea and supervised the project. M.J.A.S., M.T. and R.V. designed the experiments, R.V. and L.M.V.L. conducted most of the experiments, S.R. helped with device fabrication. All authors discussed the results and co-wrote the manuscript.

## Supplementary Material

Supplementary Information SI

Supplementary Information Response to Referees Letter (for review only VIDEO)
