# Supplementary material for: A Multiplexed Device Based on Tunable Nanoshearing for Specific Detection of Multiple Protein Biomarkers in Serum
Source: Sci Rep. 2015 May 15;5:9756. doi: 10.1038/srep09756 (PMC4432869; doi:10.1038/srep09756)
Supplement: Supplementary Information — SI [file srep09756-s1.pdf]

Supplementary Information

for

A Multiplexed Device Based on Tunable *Nanoshearing* for Specific Detection of Multiple Protein Biomarkers in Serum

Ramanathan Vaidyanathan,[1†] Lara Michelle van Leeuwen,[1†] Sakandar Rauf,[1] Muhammad J. A. Shiddiky,[1] Matt Trau[1,2]**

[1] Centre for Personalised Nanomedicine, Australian Institute for Bioengineering and Nanotechnology (AIBN), Corner College and Cooper Roads (Bldg 75), The University of Queensland, Brisbane QLD 4072, Australia

[2]School of Chemistry and Molecular Biosciences, The University of Queensland, Brisbane, QLD 4072, Australia

**Email: m.shiddiky@uq.edu.au (M. J. A. S.); m.trau@uq.edu.au (M. T.)*

[†] Authors contributed equally

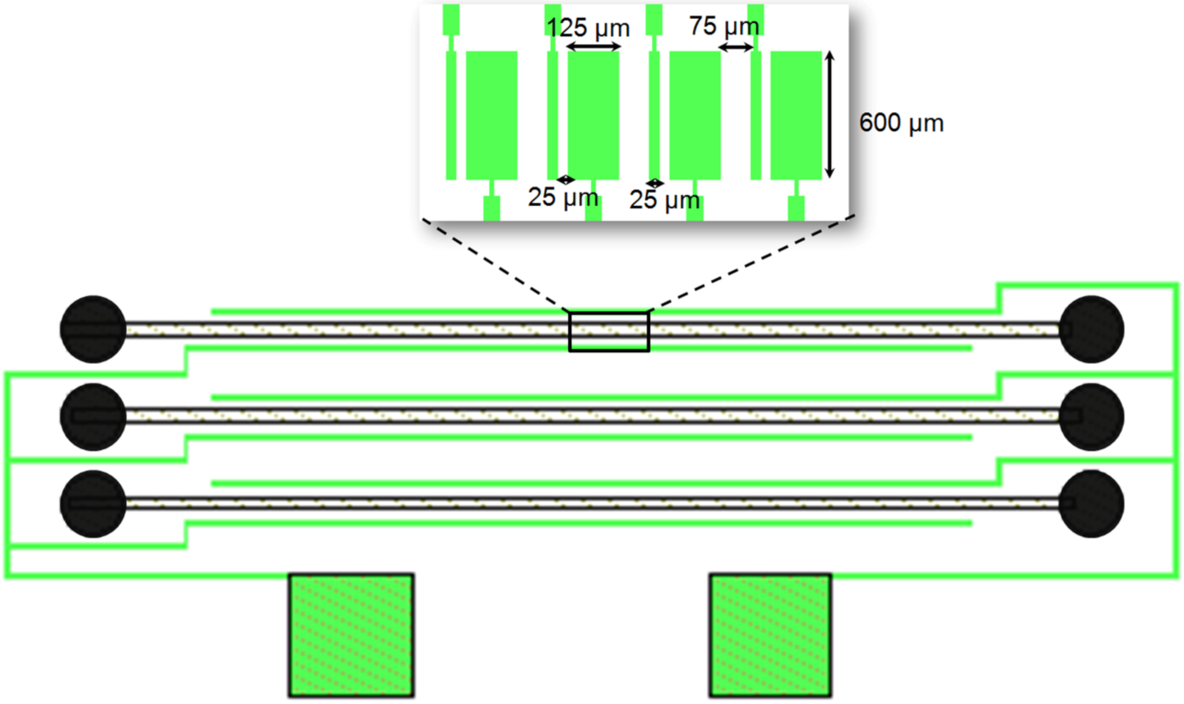

Supplementary Figure S1 | Design of multiplexed microfluidic device. Schematic of layout editor design of a multiplexed microfluidic device. The device was designed to contain three individual microfluidic channels with inlets and outlet reservoirs.

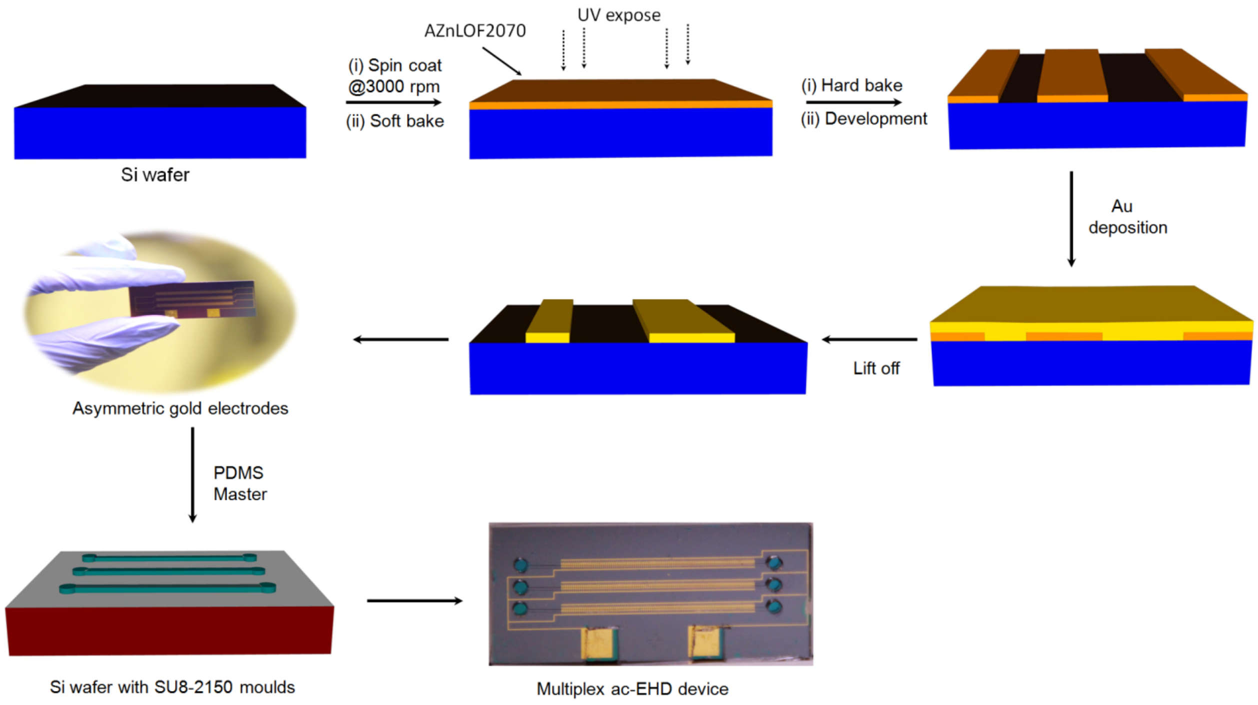

Supplementary Figure S2 | Fabrication of devices. Schematic illustration of device fabrication. The multiplexed microfluidic device contains an array of the asymmetric electrode pairs within three individual microfluidic channels.

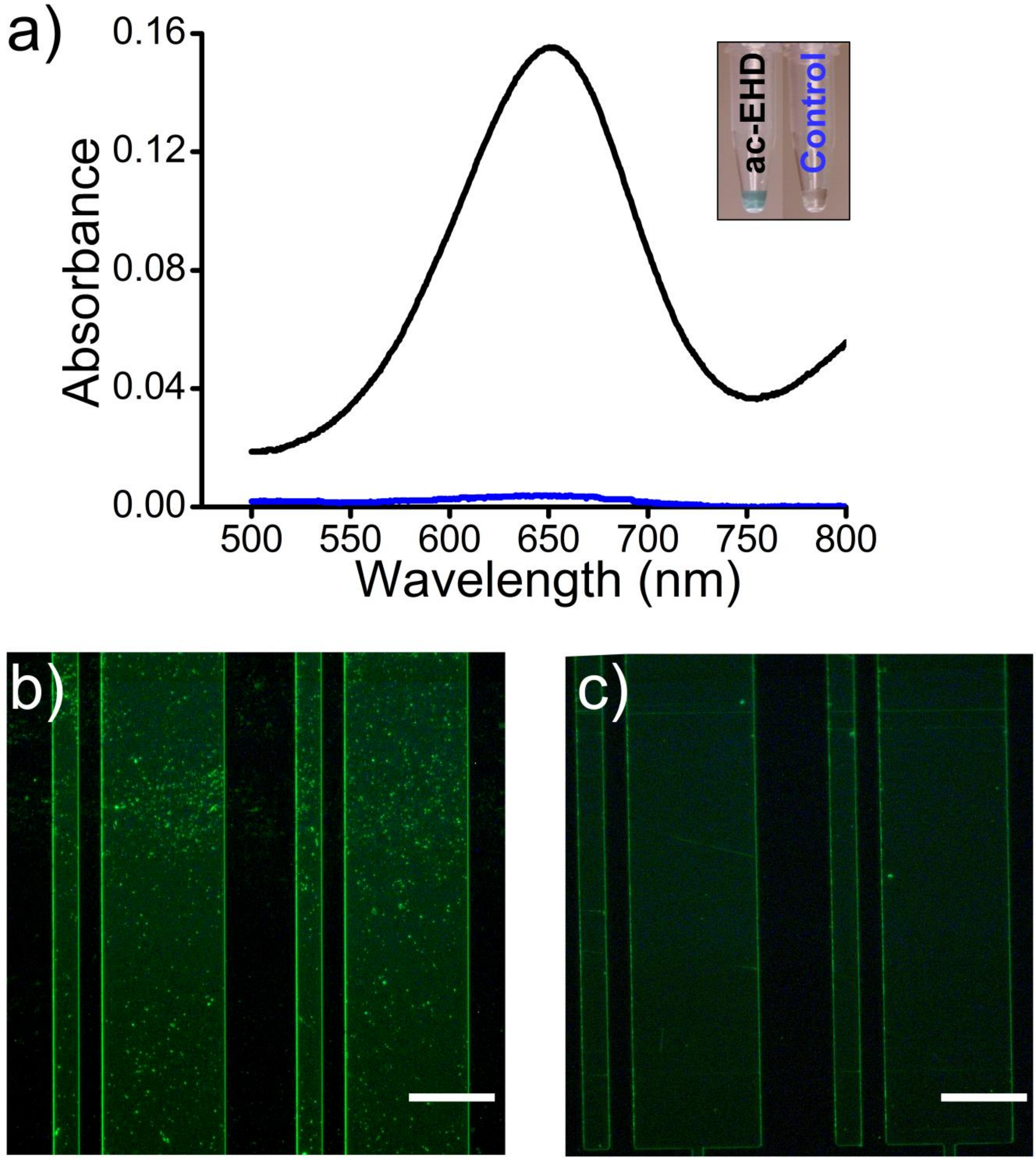

Supplementary Figure S3 | Protein detection using *nanoshearing* device. (a) UV-Vis absorbance spectra for serum samples spiked with (black; 100 pg mL^{-1}) and without (blue) HER2 under ac-EHD field strength of f = 1 kHz, V_{pp} = 100 mV. (b,c) Representative fluorescence images of the (b) detected protein (intensity: 1.73 × 10^6 (counts)) and (c) nonspecifically bound detection antibody (intensity: 1.08 × 10^3 (counts)) under ac-EHD induced fluid flow conditions. Scale bar is 100 μm.

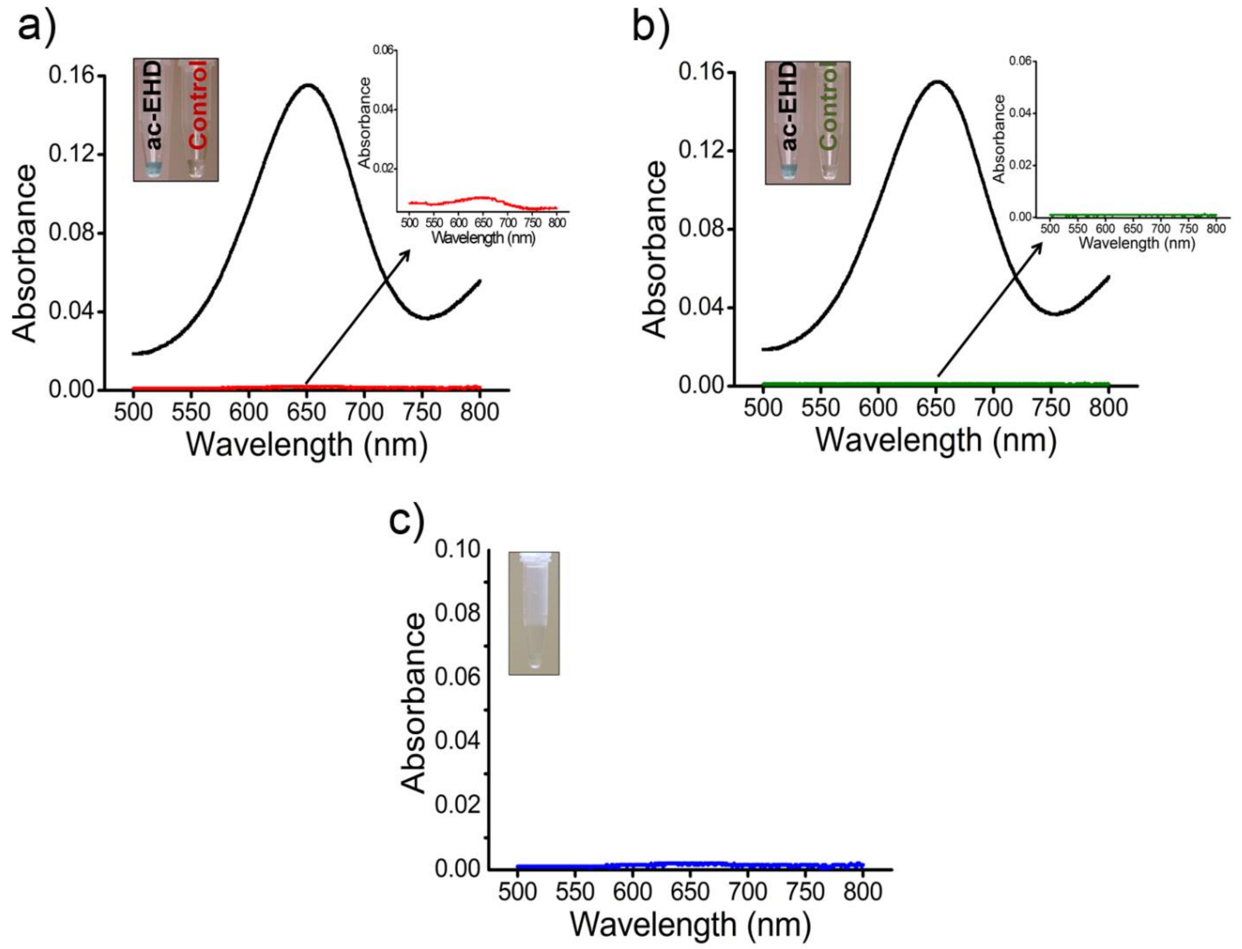

Supplementary Figure S4 | Specificity of protein capture and detection. (a, b) UV-Vis absorbance spectra of HER2 (100 pg mL^{-1}) spiked in serum driven through the devices (a) with (black) and without (red) anti-HER2 capture antibody, and (b) with (black) and without (green) FITC anti-HER2 detection antibody, under ac-EHD field strength of f = 1 kHz, V_{pp} = 100 mV. (c) UV-Vis absorbance spectra of device incubated with FITC anti-HER2 detection antibody in the absence of capture antibody and target protein.

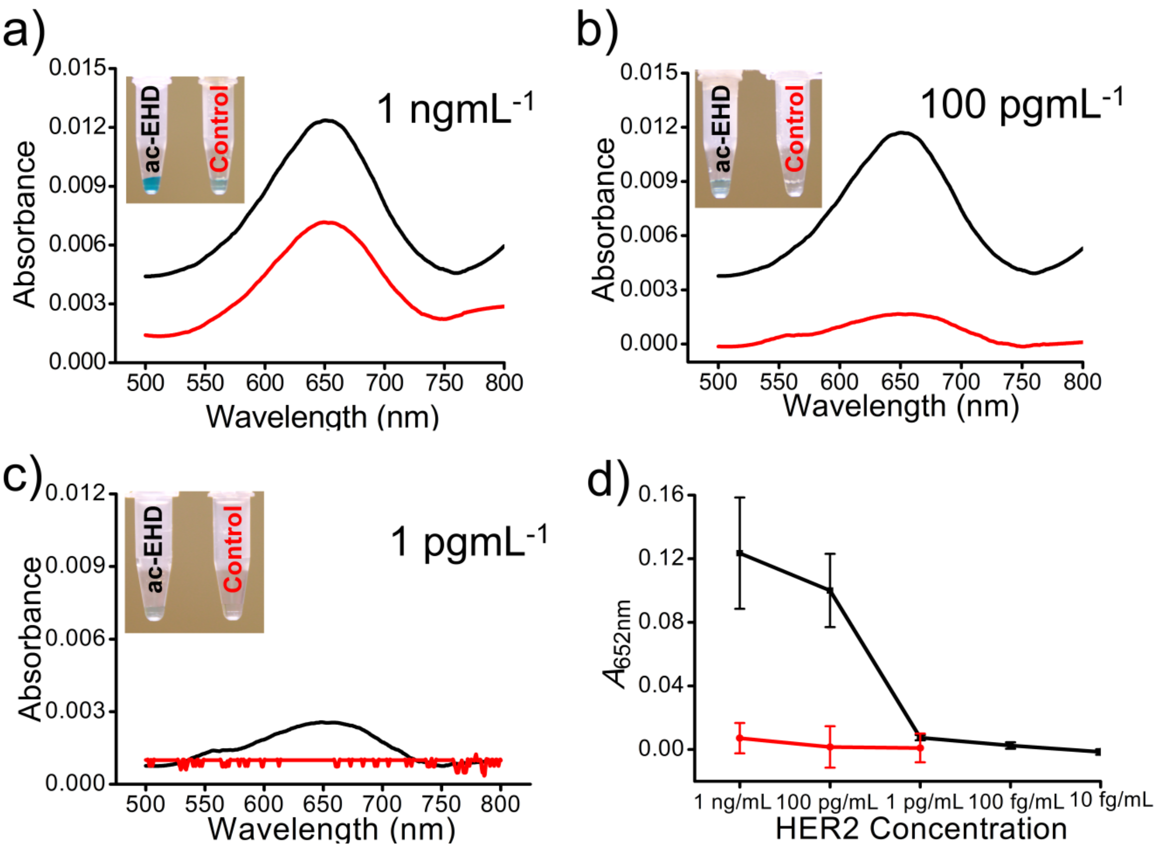

Supplementary Figure S5 | Protein detection under ac-EHD flow vs hydrodynamic flow. (a-c) UV-Vis absorbance spectra of the detected HER2 protein spiked in human serum at concentration of (a) 1 ng mL^{-1}, (b) 100 pg mL^{-1} and (c) 1 pg mL^{-1}, respectively under ac-EHD (f = 1 kHz, V_{pp} = 100 mV; black) and pressure driven flow (control; red) conditions. Pressure driven flow based devices operated under the rate of 8 μLmin^{-1} (an equivalent flow rate of that calculated based on the time required to flow 1 mL of serum sample under the given ac-EHD field). Inset shows naked eye detection of the detected HER2 protein under ac-EHD and pressure driven flow conditions. (d) Absorbance peak at 652 nm (A_{652nm}) for HER2 (1 ng mL^{-1} to 10 fg mL^{-1}) spiked in serum under ac-EHD (f = 1 kHz, V_{pp} = 100 mV; black) and pressure driven flow (control; red) conditions. Pressure driven flow based devices operated under the rate of 8 μLmin^{-1} (an equivalent flow rate of that calculated based on the time required to flow 1 mL of serum sample under the given ac-EHD field). Each data point represents the average of three separate trials *(n = 3)* and error bars represent standard error of measurements within each experiment.

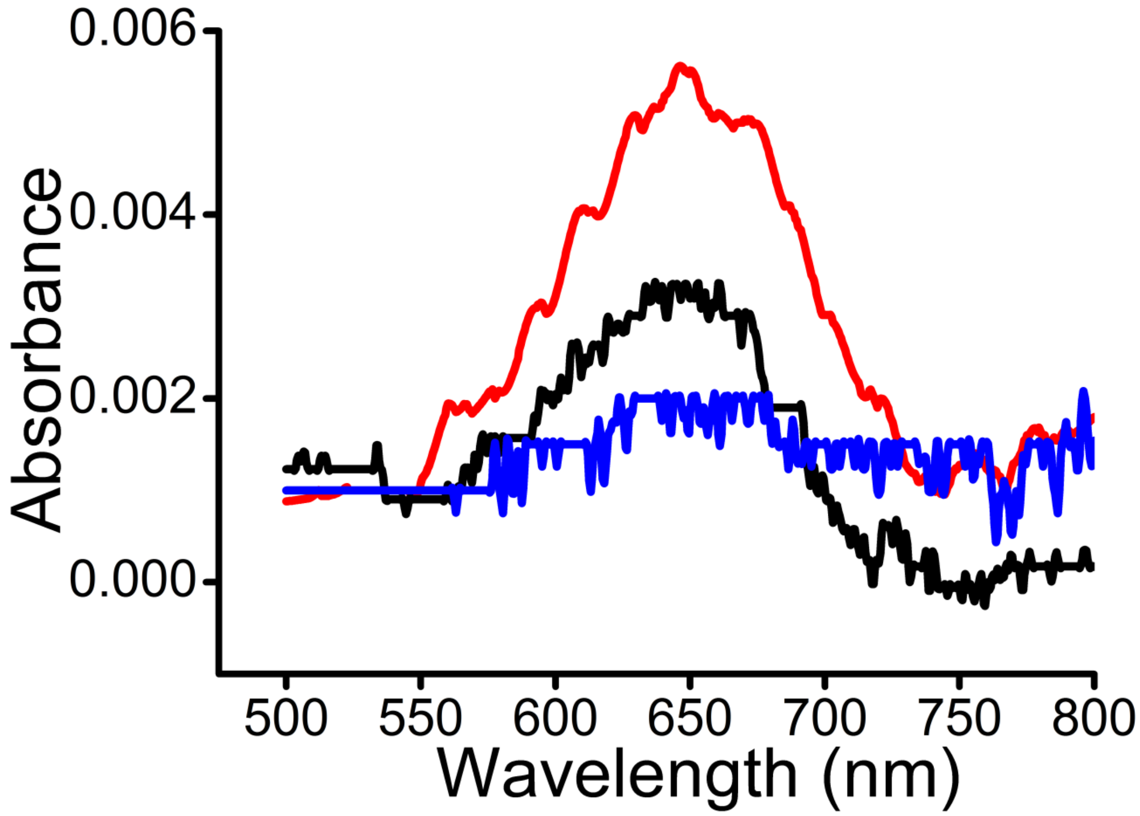

Supplementary Figure S6 | Specificity of immunocapture. UV-Vis absorption spectra of serum samples spiked with (red; 100 fg mL^{-1}) and without (black) target HER2 protein along with nonspecific PSA and IgG proteins (1 ng mL^{-1} for both cases) on anti-HER2 functionalized device. The level of background response (nonspecific adsorption of detection antibody; blue) was obtained using serum (without any specific or nonspecific proteins) on the anti-HER2 functionalized device. Data presented was obtained under the ac-EHD field strength of f = 1 kHz at V_{pp} = 100 mV.
